# Gallic Acid Crosslinked Gelatin and Casein Based Composite Films for Food Packaging Applications

**DOI:** 10.3390/polym14194065

**Published:** 2022-09-28

**Authors:** Saurabh Bhatia, Ahmed Al-Harrasi, Mohammed Said Al-Azri, Sana Ullah, Hafiz A. Makeen, Abdulkarim M. Meraya, Mohammed Albratty, Asim Najmi, Md. Khalid Anwer

**Affiliations:** 1 Natural and Medical Sciences Research Center, University of Nizwa, P.O. Box 33, Birkat Al Mauz, Nizwa 616, Oman; 2 School of Health Science, University of Petroleum and Energy Studies, Prem Nagar, Dehradun 248007, India; 3 Pharmacy Practice Research Unit, Clinical Pharmacy Department, College of Pharmacy, Jazan University, P.O. Box 114, Jazan 45142, Saudi Arabia; 4 Department of Pharmaceutical Chemistry and Pharmacognosy, College of Pharmacy, Jazan University, P.O. Box 114, Jazan 45142, Saudi Arabia; 5 Department of Pharmaceutics, College of Pharmacy, Prince Sattam Bin Abdulaziz University, P.O. Box 173, Al-kharj 11942, Saudi Arabia

**Keywords:** gelatin, casein, gallic acid, edible films, polymers, crosslinking

## Abstract

In the current work, we fabricated gelatin–casein-based edible films (GC-EFs) crosslinked with gallic acid (GA). We analyzed the physiochemical characteristics, crystallinity, thermal stability, and surface properties of the EFs using Fourier-transform infrared (FTIR) spectroscopy, X-ray diffraction (XRD), thermogravimetric analysis (TGA), and scanning electron microscopy (SEM). It was found that the edible films possessed a semi-crystalline structure. Addition of GA enhanced the thermal stability of the edible films as well as the surface properties of the films. It was found that a higher concentration of GA (4–5% *w*/*v*) significantly improved the surface properties, observed in the surface and cross-sectional examination of SEM micrographs. EFs containing higher amounts of GA showed more compact and denser structures with smoother and more homogeneous surfaces than the control samples. In addition, swelling degree (SD), thickness, water solubility (WS), moisture content (MC), and water vapor permeability (WVP) were found to be low in EFs containing more GA concentration. Mechanical parameters revealed that the Young modulus (Ym) and tensile strength (TS) increased with a rise in GA concentration, and elongation at break (EB) reduced with a rise in GA concentration. In transparency and color analysis, it was observed that GA positively affected the transparency of the edible films.

## 1. Introduction

Due to the growing environmental and human health-related concerns regarding synthetic food-packaging materials, biodegradable films including hydrocolloid, lipid and composite-based films have received great attention. These biodegradable packaging materials are considered safe as they do not contain harmful chemicals that are found in synthetic petroleum-derived polymer-based food packaging materials [1]. Additionally, these natural polymer-based films are biodegradable, edible and offer effective biological properties (antioxidant and antimicrobial) for improving quality and shelf life of packaged food [2]. Composite films made up of multiple biopolymers are preferred over single polymer-based films for their beneficial properties. Several biodegradable composites or multicomponent systems have been used so far for fabrication of these films [3,4,5]. Based on a type of biopolymer, films or coating materials are divided into three types: lipid, protein, and polysaccharide-based films. In contrast to polysaccharides and lipids, protein-based films exhibit improved mechanical and gas barrier properties [6]. Similarly, proteins are extensively used for food packaging due to characteristics such as good film-forming ability, maintenance of high nutritional value, and abundant availability [7]. Moreover, protein-based films exhibit excellent mechanical and gas barrier properties [8] as well as serving as an excellent vehicle for carrying a wide range of functional additives [9]. Physiochemical properties of proteins-based films could be improved for better performance using multiple approaches [7]. Nevertheless, protein-based films possess some limitations, such as low water vapor resistance and lesser mechanical strength, which restrict their applications [10].

Gelatin (G) is an amphoteric polymer soluble in hot water, and casein (C) is a negatively charged polymer (soluble in pH range 3–5). These edible polymers are abundantly available, biocompatible, biodegradable, possess UV light-absorption potential (preventing photo-oxidation) and are non-toxic in nature [11,12]. Additionally, both biopolymers are compatible and could be supplemented with a wide range of additives [13].

Crosslinking of these biopolymers has been proved to be an appropriate action to surmount the disadvantages possessed by protein-based polymers [14]. Nevertheless, the identification of a suitable crosslinker is critical. It is important to identify a natural, safe and edible crosslinking agent which must be effective in crosslinking functional groups of similar proteins or different proteins, to fabricate packaging material with improved, mechanical, thermal, gas barrier and water resistance properties [15]. Several synthetic aldehydes (such as formaldehyde and glutaraldehyde, carbodiimides, polyepoxy compounds, acylazide etc.) and natural crosslinking agents (such as vanillin, cinnamaldehyde, plant-derived chemical agents such as gallic acid, tannic acid, and ferulic acid) have been studied for their crosslinking effects in protein and polysaccharide-based films. A naturally occurring polyphenolic compound known as gallic acid (3,4,5-trihydroxybenzoic acid), is highly antioxidant, antimicrobial and well known for many additional health benefits [16].

Gallic acid is safe, edible and apart from its crosslinking effects it also acts as an effective preservative by preventing food oxidation and contamination by microorganism [17]. Several derivatives of gallic acid such as propyl, hexadecyl, lauryl, octyl, and tetradecyl gallate are known for their edible nature and high antioxidant effects. Thus, they can be potentially used as crosslinking agents as well as preservatives in the food industry. Gallic acid is used as a cross-linker in combination with chitosan, cellulose, and other hydrocolloids [18,19]. Films incorporated with chitosan and tuna lipid fractions crosslinked using gallic acid revealed improved permeation and mechanical characteristics [20]. Modified gallic acid crosslinked gelatin film exhibits improved mechanical, antioxidant and antibacterial properties compared with film made with conventional gelatin [21]. It is also found that gallic acid-based crosslinking of gelatin triggers a prominent reduction in the molecular flexibility of the materials, whereas the elastic modulus of the films endures at elevated temperatures [22].

Thus, previous research has demonstrated the positive effects of gallic acid on gelatin-based films. The current literature shows little information related to the effect of gallic acid on gelatin–casein composite materials. Therefore, in this current work we aimed to evaluate the effect of gallic acid on the chemical, physical, and antioxidant properties of gelatin–casein-based composite films.

## 2. Materials and Methods

### 2.1. Chemicals

Gelatin (Bovine, Type-B), casein (fat free ultra-purified, 0.2% fat), and gallic acid (pure, 98%) were procured from SRL Pvt Ltd., India, and Glycerol was procured from Sigma-Aldrich, United Kingdom.

### 2.2. Samples Preparation

Casein solution (7% *w*/*v*) was prepared by mixing with a carbonate buffer (pH 9) at 50 °C under continuous stirring. Similarly, a 3% solution of gelatin (G) was made by dissolving it in 80% glacial acetic acid at 50 °C for a duration of 30 min under continuous magnetic stirring. Both solutions were mixed, and pH was adjusted up to 7.0 along with a temperature of 50 °C. A film-forming solution of protein derived from the previous step was divided into equal parts. Later, each part of the solution was mixed with gallic acid (2–5% *w*/*w*) and glycerol (5% *w*/*w*) for 20 min at 50 °C. The resultant solution was transferred to plastic petri plates for film formation, and dried at optimum temperature for 72 h. Films were peeled from the surface of petri plates and stored for 48 h at ambient temperature and 55% RH before characterization. Visual characterization was performed initially to screen the films formed. The composition of the samples is highlighted in the Table 1. 

### 2.3. Thickness

For determining the thickness (in mm) of the fabricated EFs, a Micrometer instrument (Mitutoyo 2046F, Micrometer, Japan) was used.

### 2.4. Scanning Electron Microscopy (SEM)

Morphological examination of the EFs was conducted to examine cross-section and surface of the films using scanning microscope (SEM), JSM6510LA, Jeol, Japan. EFs were dipped in liquid nitrogen and then mounted on the metallic stub using adhesive tapes. Before taking the micrographs, all the samples were coated with gold.

### 2.5. Thermal Stability Assessment

Thermogravimetric (TGA) analysis of the EFs was carried out for assessing their thermal stability and weight loss pattern using a TA SDT-Q600 instrument, USA. Samples were analyzed at a temperature range of 25 to 600 °C with a ramp of 10 °C/min in a nitrogen-containing environment.

### 2.6. FTIR Spectra Analysis

For assessment of the chemical interaction between the EF components, FTIR analysis was carried out using an FTIR Tensor 37 instrument (InfraRed Bruker, Ettlingen, Germany). FTIR was calibrated to carry 32 scans over a wide spectral range (between 4000 and 400 cm^−1^) at room temperature.

### 2.7. X-ray Diffraction (XRD) Study

For determining the extent of crystallinity of the EFs, X-ray diffraction was performed using a D-8 Discover diffractometer (Brucker) at 2θ position ranging from 5–50° and 40 kV.

### 2.8. Color Analysis of the EFs

The color related parameters (a*, b*, ΔE*, and L*) of the prepared samples were studied by using a CR-400 chromameter instrument (Konica Minolta, Tokyo, Japan). This measurement is based on the CIELAB color system, where “L” denotes lightness, “a” represents green–red, b represents blue–yellow, and “CI” means chroma intensity. Various surface points of the EFs were analyzed during the measurement, and the mean value was calculated. The total color difference (ΔE*) between the color dimension of the corresponding samples of EFs and that of a standard plate (white) (L* = 62.7, a* = 1.6, and b* = 0.37) were calculated by using the following equation:

ΔE* = [(ΔL*)^2^ + (Δa*)^2^ + (Δb*)^2^]^1/2^ (1)

Here CI was calculated by using the equation mentioned below:

CI = (a^2^* + b^2^*)^1/2^

### 2.9. Mechanical Testing

For testing mechanical parameters such as tensile strength (TS), elongation at break (EAB), and young modulus (Ym) the Texture analyzer, Stable Microsystem Ltd. (Godalming, UK) was used.

ASTM 93 D882 standard procedure was used during this experiment [23]. EFs were sliced in rectangular shape with measurement of 20 × 50 mm. Sliced samples were assessed at a mechanical crosshead speed of 50 mm/min at an initial grip separation of 30 mm. All the readings were taken in triplicate manner and then the mean value was determined. TS (MPa) and EB (%) were calculated as per the following equations:

TS = Mxf/A

where Mxf represents maximum force at break (N) whereas the area of the EF cross-section (m^2^) is represented by A.

Elongation at breaks (EAB) was measured using the following equation:

EB = (D1 − D2)/D2 × 100% (2)

where D1 represents the initial length of the film, and D2 denotes the length (mm) of EFs at the breakage time.

### 2.10. Water Vapor Permeability (WVP)

For evaluation of WVP, we followed the protocol described by Zhou et al. [24] with slight modifications. The WVP was calculated by using equation as mentioned below:

P = (ΔW × Q)/(FA × TX ΔWP)(3)

where P = WVP (×10^−12^ g cm cm^2^ s^−1^ Pa^−1^); variation in weight of flask is represented by ΔW; mean film thickness (cm) is represented by Q; film area (cm^2^) is represented by FA; T = time (in sec.); variation in WV partial pressure (atm) at both sides of the films is represented by ΔWP.

### 2.11. Swelling Degree (SD) and Water Solubility (WS)

For determination of water solubility (WS) and swelling degree (SD), the Nouraddini et al. [25] protocol was followed with a slight modifications. Slices of the samples were prepared in a size of 20 × 50 mm and then the sample weight was determined for both WS and SD determination. For WS determination weighed samples were immersed and stirred for 1 h at 25 °C. EFs were dried (100 °C) for 12 h and WS of the samples was determined as per following equation:

WS = [(Wi − Wf)/Wi] × 100(4)

where, Wi and Wf represents the initial and final weights of the dried samples, respectively.

For SD determination films (20 × 50 mm) were initially weighed (W_1_) and transferred into flask containing distilled water for two minutes at room temperature. Later, films were transferred from the flask and excess water was removed by using the filter paper. Films were weighed (W_2_) and proportion of adsorbed water was determined in percentage as mentioned below:

SD = (W_2_ − W_1_)/W_1_ × 100 (5)

### 2.12. Moisture Content (MC) 

For MC analysis of the films, a known amount of 20 × 60 mm was placed in the plate of moisture content analyzer. The samples were subjected to drying at 105 °C for several minutes. Readings were taken until the steady dry weight was achieved. At last, weight variation was measured using the following equation:

MC = (WT_1_ − WT_2_)/WT_2_ (6)

where MC represents the moisture content in the films, WT_1_ represents the preliminary film mass (mg), and WT_2_ represents the final mass.

### 2.13. Statistical Analysis

For statistical analysis, mean plus standard deviation values were taken from the results performed in triplicates. One-way Anova of variance was used followed by Duncan’s test for evaluating the significance of variations between the mean values at 5% with 95% confidence using a statistical tool.

## 3. Results and Discussion

### 3.1. Scanning Electron Microscopy (SEM) Analysis

**Figure 1:**
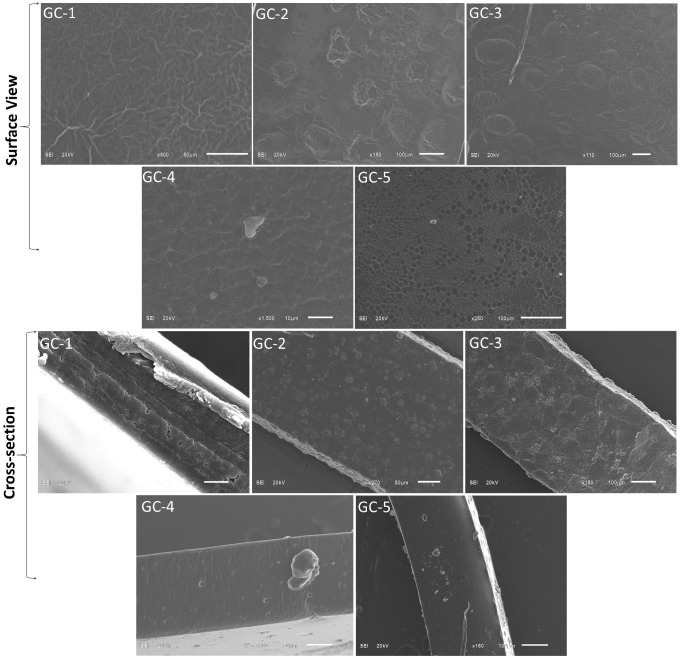
Scanning electron microscopic (SEM) analysis of edible films (GC-1, GC-2, GC-3, GC-4, and GC-5).

The surface characteristics of the films are highly associated with the physicochemical properties of the films [26]. Cross-sectional and surface views showed that GC1-GC-3 samples showed a rough, non-uniform, porous, and wrinkled surface with more deposition of particles. These features could be due to the lower concentration of GA, as its higher concentration (4–5% *w*/*v*) increased tensile strength and Y modulus to form a more compact and dense structure with smooth and homogeneous surface microstructures. The surface wrinkles that appeared in GC-3 might be associated with the greater moisture content. GC1-GC-3 samples containing (2–3% *w*/*v*) of GA showed the presence of ridges and wrinkles (Figure 1) that could be associated with the contraction (sustained during dehydration) of the samples, having more moisture content. They may also be due to the flexible and loose structures formed at lower concentration of GA. GA (4–5% *w*/*v*) crosslinked films (GC-4 and GC-5) represent stronger film networks with stronger interaction between G and C, thus preventing the formation of wrinkles on their surfaces. This could also be due to the greater cohesion of G–C matrices cross-linked by GA [27,28].

### 3.2. Film Thickness

Film thickness ranged between 35.07 and 58.17 μm (Table 2). It was found that with increase in concentration of GA, thickness of the film decreased. This could be due to the increase in the degree of crosslinking that makes the structure more compact and denser with less free volume, resulting in decrease in thickness. These findings are consistent with the previous results, where an increase in the concentration of transglutaminase resulted in a decrease in thickness of the gelatin films [29].

### 3.3. Swelling Degree

The effects of different quantities of gallic acid on the swelling degree (SD) of films are depicted in Table 2. It was found that SD of G–C films reduced with the increase in concentration of gallic acid. This could be due to the increase of cross-linking between C and G that resulted in the increase in interaction between gelatin and water molecules. However, the swelling degree of GC-1 (blank) was found to be the highest. This is due the absence of crosslinking agents, which made the polymeric matrix less dense and allowed more interaction with water molecules. Additionally, crosslinking also decreased the swelling capacities of gelatin films. Previous reports also suggested a decrease in swelling degree with the rise in the concentration of crosslinkers. Our findings are in agreement with the findings obtained from gelatin film crosslinked with glutaraldehyde and genipin, in which crosslinking significantly reduced the swelling ratio of the films [30,31,32].

### 3.4. WVP of the Edible Films

As reported earlier, poor water resistance of G–C based EFs is a major challenge that restricts its utilization in the food packaging industry [33]. It has been reported that interaction between water molecules and polymers could be reduced with cross-linking, which further impacts the water retention in the films [34]. Thus, in the present study the WVP of the GA-crosslinked G–C films were studied in detail. WVP values of blank (GC-1) and GA-crosslinked G–C films are presented in Table 2. It was found that WVP values from GC-2 to GC-5 reduced with the increase in GA concentration. This showed that water resistance of the GA-crosslinked films was enhanced in comparison to the control blank film. In the present study, film (GC-5) crosslinked with 5% *w*/*v* GA showed the lowest WVP values. Our findings were in line with the previous report that also reported a decrease in the rate of water vapor transmission with an increase in crosslinking density [35].

### 3.5. Moisture Content and Solubility

The water-resistant properties of EFs are one of their most important features, regulating the water molecules movement from the external environment to packed food product and vice versa. This feature also determines the moisture content present inside the film [36]. Moisture content is determined by the amount of water available in films (Table 2). In the present investigation, crosslinking of proteins (G-C) with GA reduced the MC and it was noticed that MC values decreased with an increase in concentration of GA. This could be due to the crosslinking effect induced by GA that made the film’s structure more compact and denser and left less space for water molecule absorption, resulting in a decrease in the moisture content.

Film solubility in water determines its biodegradability and denotes the pertinence of the film to food with more moisture content. Solubility of the G–C films decreased with an increase in GA concentration (2–5% *w*/*v*). However, the solubility of control film (GC-1) was relatively higher than GC-2-GC-5. Results obtained from the moisture content and solubility analysis showed that our results were in agreement with the previous findings [37].

### 3.6. Mechanical Assesment

The mechanical endurance of the test samples was determined using elongation at break (EB), tensile strength (TS) and young modulus (Ym) parameters. Table 3. demonstrates the dependence of TS, EB and Ym of gelatin–casein-based film on the concentration of GA. It was found that a rise in the concentration of the crosslinker (GA) increased TS and Ym values; however, it reduced EB values. In contrast, GC-1 (blank) showed lower values of TS and Ym and higher values of EB than GA-treated film. This performance of the film is ascribed to the crosslinking of the polymeric network [32,38,39]. Moreover, an increase in the concentration of GA from 2 to 5% increased TS, Y modulus, while decreasing EB values significantly (Table 3). This could be due to the effect of GA in improving intermolecular crosslinking in the protein with the formation of strong linkages due to which TS and Ym increased. Nevertheless, GA as a crosslinker induced crosslinking in the matrix to further reduce protein mobility resulting in low flexibility and EB. This response possibly is due to the decrease in deformability of the films resulting in an increase in Ym values. These findings are in agreement with previous findings [40,41].

### 3.7. Color Parameter

Color is a crucial parameter for composite films, especially for those made from different biopolymers including proteins, polysaccharides, and lipids. These composite films, due to their complex photosensitive composition, always show significant variation in color. Additionally, composite materials crosslinked with organic compounds always show color variation; thus, it is essential to assess the color characteristics of G–C crosslinked with GA. It was found that GA-crosslinked films demonstrated lower a* and b with higher L* values suggesting an increase in the light color of films when compared to blank films (GC-1). Out of all films, G–C crosslinked with GA (5%) demonstrated the highest L* values, and L* values increased with an increase in the concentration of GA (Table 4). The appearance of dark color in control films (GC-1) could be due to the protein color and oxidation reactions during film formation. A decrease in darkness could be associated with the incorporation of gallic acid which might have reduced the oxidation or organic chemical reaction, such as Maillard reaction that occurred during film formation [42].

### 3.8. Transparency

Film transparency is an important attribute that not only impacts food quality but also affects consumer acceptability [43]. In the present study, films crosslinked with GA showed less transparency than control (GC-1) and it was noticed that transparency decreased with an increase in GA concentration from (2–5% *w*/*v*) (Table 4). This could be due to the denser or more compact structural arrangement of the samples with the rise in GA concentration that might have reduced light transmittance across the films. Thus, the addition of GA impacted the appearance and light-blocking features of the films. Thus, it could also act as a UV blocking agent to reduce the deterioration of food caused by UV radiation [44]. These findings are in line with the findings obtained from the previous study [45].

### 3.9. Fourier-Transform Infrared Spectroscopy (FTIR)

**Figure 2:**
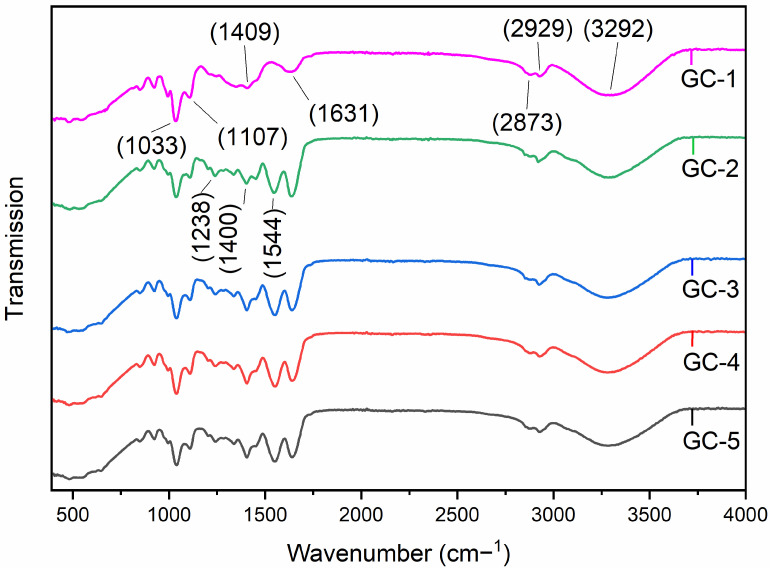
Fourier-transform infrared (FTIR) analysis of the edible films (GC-1–GC-5).

FTIR study of G–C and GA crosslinked G–C films was performed. GA is widely used due to its better stabilization proficiency of collagenous materials [11]. In the FTIR spectrum, characteristic bands were observed at 3292, 2929, 2873, 1631, 1544, 1409, 1400, 1238, 1107 and 1033 cm^−1^ positions in all samples of the edible films (Figure 2). The band position at 1631 cm-1 indicated the C=O stretching vibration, 1544 cm^−1^ showed the N-H bending vibration, and the 1238 cm^−1^ position demonstrated the stretching vibration of the C-N functional group of gelatin, respectively. In addition, the characteristic band at 3292 cm^−1^ indicated the stretching vibration of the N-H group. These FTIR findings correspond to the previous findings performed by Ichiura et al. and others [46,47].

The characteristic bands at the 2929 and 2873 cm^−1^ positions represent the stretching vibration of symmetric and asymmetric CH2 groups, respectively. These band positions indicate the existence of amino acids with a higher quantity of CH2 groups, including lysine and arginine. In addition, bands observed between 1400–1000 cm^−1^ indicate the stretching vibration of carbonyl groups of casein, and bands at 1600–1500 cm^−1^ indicate the amide (NHCO) stretching. These findings are consistent with previous results [48]. A little difference observed in the spectrum of the GC-1 sample from the others could be due to the absence of gallic acid. In addition, an increasing concentration of gallic acid had no prominent effect on the physiochemical characteristics of the fabricated edible films.

### 3.10. Thermogravimetric Analysis (TGA)

**Figure 3:**
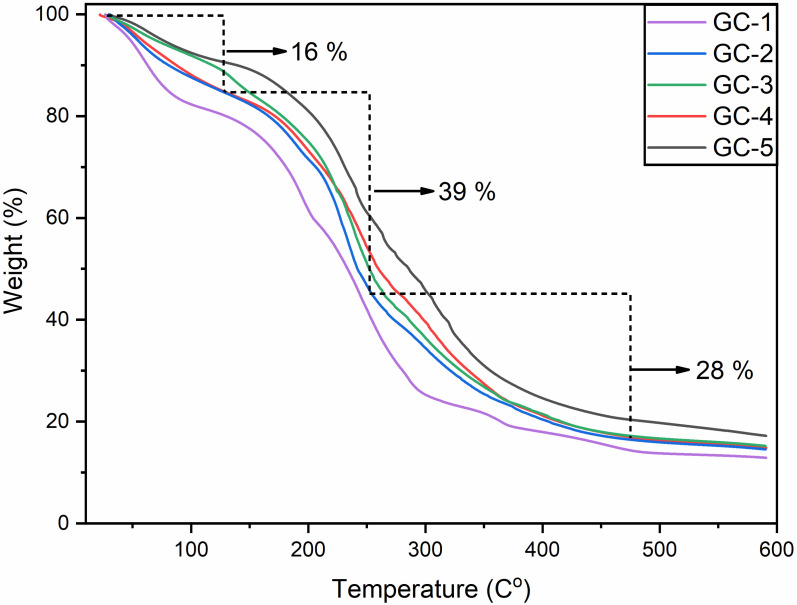
Thermogravimetric analysis of the edible films containing varied concentration of gallic acid; GC-1, GC-2, GC-3, GC-4, and GC-5.

Thermogravimetric analysis (TGA) was carried out to investigate thermal stability of G-C films crosslinked with and without GA, as shown in Figure 3. All samples demonstrated three weight loss cycles with residual mass at 600 °C. Initial weight loss observed among all samples could be due to the evaporation of water content. This weight loss started from room temperature and the final temperature was around 130 °C. The next phase of weight loss could be associated with the thermal degradation of protein with low molecular weight. The final temperature in this weight loss was around 230 °C. The last stage of weight loss was associated with the thermal decomposition of large-size protein fractions. The estimated final temperature for the thermal degradation at this stage was around 480 °C. It was found that GA increased the thermal stability of the edible films relative to control. This could be due to the stabilization of the G–C matrix with the addition of GA, as GA addition resulted in the increase in new covalent bond formation due to crosslinking and noncovalent interactions between GA and the G–C matrix. This could also be due to the changes in crystallinity of the films crosslinked with GA [49]. These findings, based on incorporation of GA in G–C films and improvement of thermal stability, were found to be similar to the findings observed by Bigi et al. [50]. GA could also be detrimental in changing the crystallinity of the G–C matrix and may encourage new interaction among the hydroxyl groups of casein with the carboxyl and amino group of gelatin [51]. Thus, such GA-dependent changes need more thermal energy for disintegration of the crystals and decreasing of the interactions, offering more thermal stability to the films [33,52].

### 3.11. X-ray Diffraction (XRD) Analysis

**Figure 4:**
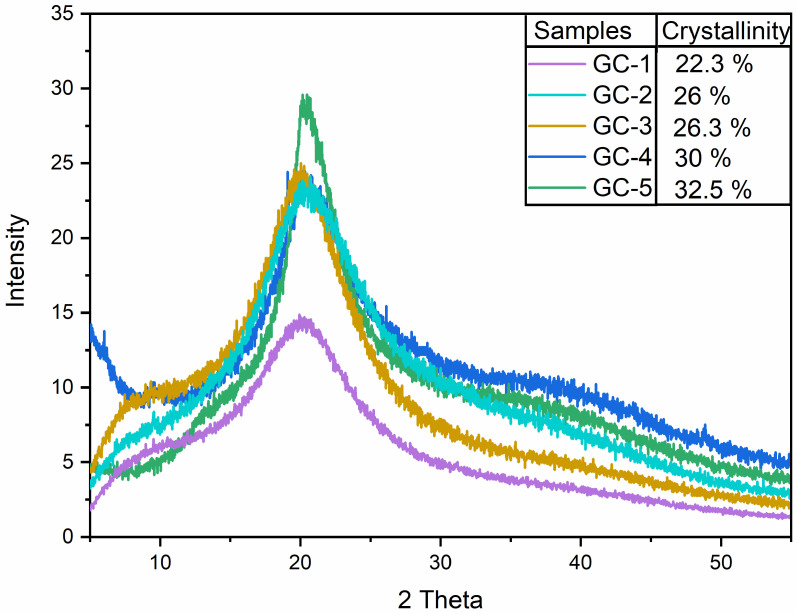
XRD analysis of the gallic acid-added gelatin–casein-based edible films; GC-1, GC-2, GC-3, GC-4, and GC-5.

The crystalline nature of G–C films was examined using XRD, and the findings are shown in Figure 4. Findings obtained from the XRD plot of all samples showed a broad peak at 20° of 2θ position which could be ascribed to the semi-crystalline structure [53,54]. Similar findings have been reported in previous studies in which gelatin/casein is crosslinked with other polymers [55,56]. Incorporation of GA promotes covalent bond formation via inhibiting the restoration of the triple helix structure. The difference in peak intensities could be associated with the difference in concentration of GA in the samples. This study is in agreement with the previous findings which found no impact of gallic acid on structural configuration of the triple helix of the collagen [57]. However, the earlier study suggested that tannic acid showed more affinity to casein in comparison to gallic acid; thus, it further requires comparative analysis to study the comparative effect of crosslinkers over the present composite material [58].

## 4. Conclusions

In this study gelatin and casein-based composite films were fabricated using gallic acid as a natural crosslinker. After examining the physicochemical, mechanical, barrier, transparency, and surface properties of gallic acid crosslinked gelatin–casein-based edible film, we found that GA addition positively impacted the film’s properties. SEM analysis revealed that GA crosslinked films (GC-4 and GC-5) exhibited better surface microstructure, and thermogravimetric investigation revealed that GA addition enhanced the thermal stability of the film. A higher concentration of GA decreased film thickness, swelling degree, water solubility, water vapor permeability, and moisture content. A decrease in values of these parameters is considered as favorable. In mechanical parameters, GA addition (higher concentration) improved the TS and Ym while it decreased the EB values. Furthermore, the addition of GA decreased the transparency of the films. We concluded that GA addition improves the characteristics of the gelatin–casein-based films.

## Figures and Tables

**Table 1:** Composition of the samples.

Codes	Chemical Composition of the Edible Films
GC-1	C + G + Gly
GC-2	C + G + Gly + GA (2%)
GC-3	C + G + Gly + GA (3%)
GC-4	C + G + Gly + GA (4%)
GC-5	C + G + Gly + GA (5%)

All of the film components were dissolved in weight by volume (*w*/*v*) formula. Casein: C (7%), gelatin: G (3%), glycerol: Gly (5%), gallic acid: GA (2–5%).

**Table 2:** Thickness, swelling degree (SD), water solubility (WS), water vapor permeability (WVP), and moisture content (MC) of G–C and G–C crosslinked films.

Sample Codes	Thickness (μm)	SD (%)	WS (%)	WVP (×10–12 g⋅cm/cm^2^⋅s⋅Pa)	MC (%)
GC-1	58.17 ± 1.2 ^a^	118.1 ± 4.21 ^a^	89 ± 3.11 ^a^	3.89 ± 0.03 ^a^	13.11 ± 0.21 ^a^
GC-2	51.17 ± 1.1 ^b^	93.1 ± 4.35 ^b^	78 ± 2.67 ^b^	3.01 ± 0.01 ^b^	11.67 ± 0.23 ^b^
GC-3	47.71 ± 1.7 ^c^	84.3 ± 1.73 ^c^	67 ± 3.24 ^c^	2.58 ± 0.01 ^c^	10.55 ± 0.17 ^c^
GC-4	41.22 ± 2.6 ^d^	69.1 ± 3.41 ^d^	54 ± 2.12 ^d^	1.75 ± 0.02 ^d^	9.11 ± 0.11 ^d^
GC-5	35.07 ± 1.7 ^e^	61.2 ± 2.17 ^e^	51 ± 1.19 ^d^	1.17 ± 0.01 ^e^	8.22 ± 0.16 ^e^

Composite film samples. GC-1, GC-2, GC-3, GC-4, and GC-5. Results expressed as mean ± standard deviation. Mean values in the same column with different superscript letters (^a–e^) are significantly different (*p* < 0.05).

**Table 3:** Mechanical properties of gelatin, and casein crosslinked films.

Sample Codes	EB (%)	TS (MPa)	Ym
GC-1	37.21 ± 1.22 ^a^	3.06 ± 0.01 ^d^	21.33 ± 2.01 ^d^
GC-2	31.22 ± 1.01 ^b^	5.02 ± 0.02 ^c^	29.13 ± 2.17 ^c^
GC-3	17.11 ± 1.2 ^c^	7.24 ± 0.01 ^b^	43.02 ± 1.3 ^b^
GC-4	15.32 ± 0.2 ^c^	7.11 ± 0.01 ^b^	48.15 ± 2.1 ^a^
GC-5	10.11 ± 0.3 ^d^	9.21 ± 0.03 ^a^	51.17 ± 3.1 ^a^

TS: tensile strength, EB: elongation at break, and Ym: young modulus. Results are presented as mean values along with values of standard deviation. Superscript letters (^a–d^) represent differences among mean values (*p* < 0.05).

**Table 4:** Transparency and color analysis and of the EFs.

Sample Codes	L	A*	B*	△E*	CI*	Transparency
GC-1	35.05 ^e^	8.21 ^a^	15.03 ^a^	31.98641 ^a^	15.07021 ^a^	3.121 ± 0.01 ^a^
GC-2	47.11 ^d^	5.11 ^b^	12.11 ^b^	19.82917 ^b^	12.18457 ^b^	2.811 ± 0.02 ^b^
GC-3	56.22 ^c^	3.07 ^c^	8.01 ^c^	10.12526 ^c^	8.41988 ^c^	2.423 ± 0.03 ^b^
GC-4	61.34 ^b^	3.62 ^c^	5.11 ^d^	5.32894 ^d^	5.280233 ^d^	2.110 ± 0.01 ^b^
GC-5	68.13 ^a^	1.18 ^d^	2.02 ^e^	5.690677 ^d^	3.517134 ^e^	1.910 ± 0.02 ^c^

A*: green-red color, B*: blue-yellow color, CI*: chroma intensity, L: lightness, ΔE*: overall color variation. Results are presented as mean values along with values of standard deviation. Superscript letters (^a–e^) represent differences among mean values (*p* < 0.05).

## Data Availability

Not applicable.
